# Septic shock in a cohort of patients from the northeast of France: a preliminary epidemiological study, EPISS group

**DOI:** 10.1186/cc9632

**Published:** 2011-03-11

**Authors:** J Quenot, A Pavon, C Binquet, F Kara, O Martinet, JC Navellou, D Barraud, J Cousson, JF Poussel

**Affiliations:** 1 University Hospital Bocage, Dijon, France; 2 Faculté de Médecine, Dijon, France; 3 Centre Hospitalier, Haguenau, France; 4 Nouvel Hôpital Civil, Strasbourg, France; 5 Centre Hospitalier Universitaire, Besancon, France; 6 Hôpital Central, Nancy, France; 7 Centre Hospitalier, Intensive Care, Reims, France; 8 Centre Hospitalier, Intensive Care, Metz, France

## Introduction

Incidence of septic shock in France ranges from 8 to 10% among patients admitted to intensive care. Mortality at 28 days is 55 to 60% [1]. We aimed to investigate epidemiology, treatment and mortality of patients with septic shock further to the Surviving Sepsis Campaign international guidelines [2].

## Methods

A prospective, multicentre, observational cohort study supported by the Collège Interrégional des Réanimateurs du Nord-Est (CIRNE) including 14 ICUs in 10 university or nonacademic hospitals. Inclusion criteria were: patients presenting with documented/suspected infection requiring initiation of vasopressor amines despite adequate vascular filling, with at least one of the following hypoperfusion criteria: metabolic acidosis (base excess ≥5 mEq/l or alkaline reserve <18 mEq/l or lactate ≥2.5 mmol/l); oliguria/renal insuf-ficiency (< 0.5 ml/kg/hour for 3 hours or elevation >50% of baseline creatinine); or hepatic dysfunction (AST or ALT >500 IU/l or bilirubin >20 mg/l (34 μmol/l)). Quality control was performed by the Dijon Clinical Investigation Center (INSERM).

## Results

Mean inclusion was 80 patients/month for all centres. We analysed the first 350 patients with validated files of 876 patients included up to 1 December 2010. Mean age was 69 ± 13 years, 66% men. Indication for admission was medical in 84%. Mean SAPS II score was 60.9 ± 21.8, mean SOFA score at time of shock was 11.7 ± 3.5. Sepsis was mainly of pulmonary (45.7%), digestive (19.4%), or urinary (11.1%) origin, with 23.8% other causes. Sepsis was mainly community-acquired (63.7%) and was documented in 67% (234/350), of which 53.4% were Gram-negative bacilli, 30.3% Gram-positive cocci and 16.3% others. Replacement techniques used were: invasive mechanical ventilation (82.6%), continuous dialysis (31.1%) and discontinuous dialysis (19.7%). Activated protein C was used in 17 patients (5%) and hydrocortisone hemisuccinate in 238 (68.6%). Mortality was 49.1% in intensive care, 58.8% in-hospital.

## Conclusions

Our findings raise hope of improved knowledge of epidemiology and management of septic shock in intensive care patients, and should have a beneficial effect on prognosis.
